# Cell-Free Supernatant of *Odoribacter splanchnicus* Isolated From Human Feces Exhibits Anti-colorectal Cancer Activity

**DOI:** 10.3389/fmicb.2021.736343

**Published:** 2021-11-11

**Authors:** Byeong Seob Oh, Won Jung Choi, Ji-Sun Kim, Seoung Woo Ryu, Seung Yeob Yu, Jung-Sook Lee, Seung-Hwan Park, Se Won Kang, Jiyoung Lee, Won Yong Jung, Young-Min Kim, Jae-Ho Jeong, Ju Huck Lee

**Affiliations:** ^1^Korean Collection for Type Cultures, Biological Resource Center, Korea Research Institute of Bioscience and Biotechnology, Jeongeup, South Korea; ^2^Korean Bioinformation Center, Korea Research Institute of Bioscience and Biotechnology, Daejeon, South Korea; ^3^Department of Food Science and Technology, and Bio-energy Research Center, Chonnam National University, Gwangju, South Korea; ^4^Department of Microbiology, Chonnam National University Medical School, Gwangju, South Korea

**Keywords:** gut microbiota, *Odoribacter splanchnicus*, colorectal cancer, cell-free supernatant, apoptosis, murine model

## Abstract

The gut microbiota (GM) has been shown to be closely associated with the development of colorectal cancer (CRC). However, the involvement of GM is CRC has mainly been demonstrated by metagenomic profiling studies showing the compositional difference between the GM of healthy individuals and that of CRC patients and not by directly studying isolated gut microbes. Thus, to discover novel gut microbes involved in CRC, we isolated the GM from the feces of healthy individuals and evaluated its anti-CRC activity *in vitro* and *in vivo*. After GM isolation, cell-free supernatants (CFSs) were prepared from the isolated gut microorganisms to efficiently screen a large amount of the GM for anti-proliferative ability *in vitro*. Our results showed that the CFSs of 21 GM isolates had anti-proliferative activity against human colon cancer HCT 116 cells. Of these 21 GM isolates, GM07 was chosen for additional study because it had the highest anti-cancer activity against mouse colon cancer CT 26 cells *in vitro* and was further evaluated in a CT 26 allograft mouse model *in vivo*. GM07 was identified as *Odoribacter splanchnicus* through phylogenetic analysis based on 16S rRNA gene sequencing. Further investigation determined that the CFS of *O. splanchnicus* (OsCFS) induced anti-proliferative activity *via* apoptosis, but not cell cycle arrest. Moreover, GC/MS analysis suggested that the putative active molecule in OsCFS is malic acid. Finally, in the CRC mouse model, peri-tumoral injection of OsCFS significantly decreased CRC formation, compared to the control group. Altogether, these findings will provide valuable information for the discovery of potential probiotic candidates that inhibit CRC.

## Introduction

Colorectal cancer (CRC), a cancer of the gastrointestinal tract, is one of the most common forms of cancer with increasing incidence and high mortality worldwide (Jemal et al., 2010). Many factors, including genetic, environmental, diet, and lifestyle factors, contribute to the risk of CRC (Marshall, 2008). In addition, a large number of intestinal bacteria, called gut microbiota, are closely associated with tumorigenesis in CRC (Gagnière et al., 2016). Indeed, many reports demonstrating changes in the diversity of gut microbiota in CRC patients have suggested that gut dysbiosis, or an imbalanced gut microbiome, is a major factor for colon carcinogenesis (Nakatsu et al., 2015; Zou et al., 2018). For example, the dominant abundance of the phyla Bacteroidetes, Proteobacteria, and Fusobacteria was determined in CRC patients compared to that in healthy individuals; in contrast, the phylum Firmicutes was abundant in healthy individuals (Zackular et al., 2013; Liu et al., 2020). However, the involvement of gut microbiota in CRC has mostly been demonstrated through next-generation sequencing techniques, such as 16S rRNA-based metagenomics, showing the diversity and compositional differences of the gut microbiome in CRC patients (Yachida et al., 2019). Despite metagenomic studies suggesting that many different gut microbes are related to CRC, direct evidence of the involvement of the isolated gut microbiota in CRC is limited, except for lactic acid bacteria (LAB). Hence, we focused on the discovery of novel gut microbes, residing commensally in the gut of healthy individuals and possibly suppressing CRC development, through experimental studies using isolated gut microbes.

Probiotics are defined as live microorganisms that, when administered in sufficient amounts, confer a health benefit to the host, and LAB, including *Lactobacillus* spp. and *Bifidobacterium* spp., are most commonly used as probiotics (Pool-Zobel et al., 1996; Rowland et al., 1998). Over the past few decades, probiotics have been reported to inhibit CRC development through several mechanisms, including modulation of the host immune response, production of short-chain fatty acids (SCFAs) or bioactive molecules that induce apoptosis of colon cancer cells (Konishi et al., 2016; Sánchez-Alcoholado et al., 2020), and competition with CRC-associated pathogenic microbiota, such as *Bacteroides* spp., *Clostridium* spp., and *Fusobacterium* spp. in the intestinal environment (Rafter et al., 2007; Lawrence et al., 2020). Previous studies have reported that administration of LAB, such as *Lactobacillus kefiranofaciens* and *Lactobacillus casei*, could modulate the host immune system response by activating immune cells to eliminate cancer cells in the host (Galdeano and Perdigón, 2006; Vinderola et al., 2006). In addition, co-administration of vitamin K1 and *Lactobacillus rhamnosus* GG showed enhanced anti-proliferative efficacy due to the induction of apoptosis and cell cycle arrest, leading to the inhibition of proliferation in colon adenocarcinoma cells (Orlando et al., 2016). However, there may still be several unidentified gut microbes that could control CRC development, as has been suggested by metagenomic studies. Therefore, the discovery of novel gut microbiota with the potential for use as probiotics that enhance the protective effect against CRC is needed for better treatment strategies.

Inflammatory bowel disease (IBD), such as ulcerative colitis and Crohn’s disease (CD), are the main causes leading to CRC development; they cause alterations in the composition of gut microbiota in the host intestinal environment (Kulaylat and Dayton, 2010; Khor et al., 2011). Compared to healthy individuals, patients with IBD show a decrease in diversity, a lower proportion of Firmicutes at the phylum level, a higher proportion of Gammaproteobacteria at the class level, and a lower proportion of *Lachnospiraceae* and *Ruminococcaceae* at the family level (Joossens et al., 2011). Moreover, the proportions of the genera *Roseburia*, *Faecalibacterium*, and *Odoribacter* were reduced (Morgan et al., 2012). *Roseburia* spp. and *Faecalibacterium* spp. have been considered to be bioindicators of human health; the risk of CRC or IBD is increased when their abundance levels are lower than those in healthy individuals (Ferreira-Halder et al., 2017; Lawrence et al., 2020). Additionally, it was shown that the abundance of genus *Odoribacter* was decreased in the patients of CRC and polyps as compared with the control group (Fang et al., 2021).

*Odoribacter splanchnicus* is a Gram-negative, obligately anaerobic, non-spore-forming commensal bacteria residing in the human intestinal tract, which produces SCFAs, such as acetate, propionate, and butyrate (Werner et al., 1975). According to previous reports, SCFAs produced by the gut microbiota can induce anti-proliferation, apoptosis, and cell cycle arrest in colon carcinoma cells (Hinnebusch et al., 2002). In addition, outer membrane vesicles (OMVs) produced by *O. splanchnicus* can improve the intestinal environment by decreasing the pro-inflammatory cytokine interleukin-8 (IL-8) response and inducing the anti-inflammatory cytokine IL-10 in HT-29 enterocytes (Hiippala et al., 2020). However, despite the potential of the anti-CRC effect *O. splanchnicus* demonstrated by metagenomic data and cell-based results, there have been no relevant studies showing a direct correlation between CRC and *O. splanchnicus*. Thus, whether *Odoribacter* spp. is a candidate for anti-CRC activity should be verified by experimental evidence.

Metagenomic analysis has suggested that some commensal gut microbiota may have potential health benefits. However, verification of these potential roles at the species level remains unexplored. In this study, we experimentally evaluated whether the gut microbiota isolated from healthy individuals could control CRC development *in vitro* and *in vivo*. First, the gut microbes were isolated from fecal samples of healthy individuals, and cell-free supernatants (CFSs) were prepared to efficiently screen anti-CRC activity in a cell-based system. Thereafter, the anti-tumor effect was evaluated *in vitro,* and *O. splanchnicus* was selected for future studies. We demonstrated that the CFS of *O. splanchnicus* (OsCFS) induced apoptotic cell death in colon cancer cells and demonstrated that the anti-CRC molecule of OsCFS showed non-proteinaceous properties that were heat-stable and protease-insensitive. Moreover, the potential active molecule of OsCFS was speculated to be malic acid in the GC/MS analysis. Finally, OsCFS was found to inhibit CRC development *in vivo* in a mouse allograft model of CRC. Altogether, our study findings suggest that *O. splanchnicus* is a promising new probiotics for controlling CRC development.

## Materials and Methods

### Cell Culture

The colon cancer cell lines HCT 116 and CT 26 were obtained from the Korean Collection for Type Cultures (Jeongeup, Republic of Korea). In addition, the normal colon cell line CCD 841 CoN was purchased from the American Type Culture Collection (Cat #CRL-1790, VA, United States). The cells were maintained in Dulbecco’s modified Eagle’s medium (DMEM; Gibco, NY, United States) supplemented with 10% fetal bovine serum (FBS; Gibco, NY, United States), 200U/ml penicillin, and 100μg/ml streptomycin. All cells were cultured at 37°C in a humidified atmosphere of 5% CO_2_.

### Mice

Male BALB/c mice (aged 6–8weeks) were obtained from KOSA BIO Inc. (Seongnam, Republic of Korea). All mice were raised under specific pathogen-free conditions, with a temperature of 22±2°C, humidity of 55±5%, and a 12-h light/dark cycle. Commercial rodent chow and water were supplied *ad libitum*. All experimental procedures were approved by the Institutional Animal Care and Use Committee of the Korea Research Institute of Bioscience and Biotechnology (approval number: KRIBB-AEC-18063), and all animals were cared for according to the guidelines for animal experiments of the Korea Research Institute of Bioscience and Biotechnology.

### Fecal Gut Microbiota Isolation and Identification

The gut microbiota was isolated from fecal samples of 100 healthy individuals who had not taken any medicine and had a normal BMI (IRB: P01-201702-31-007). Fecal samples were collected from the Seoul National University Bundang Hospital (Seongnam, Republic of Korea). The isolation and culture of bacteria from fecal samples were performed in an anaerobic chamber (Coy Laboratory Products, Grass Lake, MI, United States) filled with 86% N_2_, 7% CO_2_, and 7% H_2_. One gram of one fecal sample was suspended in 10ml of sterilized 0.85% saline solution, followed by serial dilution up to 10^−6^. A 100μl of the diluted sample was spread and cultivated on tryptic soy agar (BD, NJ, United States) supplemented with 5% sheep blood (TSAB). After incubation, 50–100 colonies per plate were grown. To isolate gut microbes, 20 single colonies per plate were randomly transferred onto new TSAB agar plates under anaerobic conditions. To identify a total of 2,000 isolated colonies from 100 fecal samples, 16S rRNA sequence was amplified by polymerase chain reaction from cell suspensions using universal 16S rRNA bacterial primers: 27F (5'-AGA GTT TGA TCM TGG CTC AG-3') and 1492R (5'-TAC GGY TAC CTT GTT ACG ACT T-3'). Then, the amplified 16S rRNA gene was sequenced with universal primers: 785F (5'-GGA TTA GAT ACC CTG GTA-3') and 907R (5'-CCG TCA ATT CMT TTR AGT TT-3') (Macrogen, Inc.). To confirm taxonomic position of the isolate, BLAST search was performed between the sequenced 16S rRNA gene and the sequences obtained from the EzBioCloud database^1^ (Yoon et al., 2017). Thereafter, the isolate was preserved at −80°C in 10% (v/v) skim milk solution.

### CFS Preparation

To prepare CFSs of the gut microbiota, we modified a previously described method (Ma et al., 2010; Escamilla et al., 2012). Briefly, the isolated gut microbes were grown on each TSAB plates in an anaerobic chamber. After cultivation, the colonies of the gut microbe were suspended in 1ml of PBS and adjusted to an optical density of 0.5 at 600nm (OD_600_). Thereafter, 500μl of the gut microbe suspension was transferred into 20ml of reduced reinforced clostridial medium (RCM; MB cell, Seoul, Republic of Korea) broth which was best suitable for the liquid culture of most the isolated GMs, followed by anaerobic incubation at 37°C for 48h. After incubation, the cells were removed by centrifugation at 6000×*g* for 30min, and the supernatant was harvested. To counter the side effects of pH, neutralization was adjusted using 1M NaOH at pH 6.8, which is the same as the pH value of fresh RCM broth, and the neutralized CFSs were filtered through a syringe filter with a 0.22-μm hydrophilic polyethersulfone membrane. The CFSs were stored at −80°C until further use.

### Anti-proliferative Activity Measurement Using an MTT Assay

To determine the anti-proliferative activity of the CFSs, an MTT assay was performed. HCT 116, CT 26, or CCD 841 CoN cells were seeded at 1×10^4^, 3×10^3^, and 2×10^3^ cells per well in 96-well plates, respectively. The medium was replaced with fresh medium containing 10% (v/v) of CFS, followed by incubation at 37°C for 72h. Cells treated with 10% (v/v) of RCM were used as controls. At the end of the experiments, the medium was replaced with 100μl of serum-free medium containing 10% MTT solution (5mg/ml) in each well. After 2h of incubation at 37°C, the medium was gently removed, and the colored formazan product was dissolved in 100μl of 40mM HCl-isopropanol. Cell viability was measured by reading the absorbance at 595nm using a microplate reader (Multiskan FC; Thermo Fisher Scientific, MA, United States). Anti-proliferative activity was calculated using the following equation: Anti-proliferative activity (%)=100 – A_treatment_/A_control_×100.

### Crystal Violet Staining Assay

To visualize the anti-proliferative activity of OsCFS, HCT 116 cells were seeded at 5×10^4^ cells per well in 6-well plates and stabilized overnight. After the removal of the medium, fresh medium containing 10% of RCM or OsCFS was added to duplicate plates, followed by incubation for 72h at 37°C. The cells in one plate were gently rinsed twice in DPBS, fixed with 500μl of 4% paraformaldehyde for 10min, and stained with 500μl of 1% crystal violet staining solution for 20min at room temperature. After staining, the cells were gently washed twice in DPBS, followed by air dry. Thereafter, the colonies were observed. The remaining plates were used for viable cell counting.

### Flow Cytometry for Apoptosis and Cell Cycle Analysis

Apoptosis was evaluated by FITC-conjugated Annexin V and propidium iodide (PI) flow cytometry using a commercial kit (Thermo Fisher Scientific, MA, United States), according to the manufacturer’s instructions. Briefly, HCT 116 cells were seeded at 5×10^5^ cells in 100-mm cell culture dishes and stabilized overnight. The next day, the cells were treated with 10% (v/v) of OsCFS mixed with fresh medium for 72h. After the OsCFS treatment, the cells were harvested, washed twice with PBS, and centrifuged (200×*g*, 4°C, 5min), and the cell pellets were resuspended in 500μl of binding buffer according to the manufacturer’s instructions. Next, the cells were labeled with 5μl of fluorochrome-conjugated Annexin V added to 100μl of the cell suspension and incubated for 10min at room temperature. After incubation, the cells were washed with binding buffer and resuspended in 200μl of binding buffer. Finally, the cells were stained with 5μl of PI solution and analyzed using an Attune NxT Flow Cytometer (Thermo Fisher Scientific, MA, United States) for the detection of Annexin V- and PI-positive subpopulations. Cells treated with RCM were used as negative controls.

For the cell cycle assay, HCT 116 cells were prepared using the cell harvesting step as described above. The harvested cells were washed once with PBS and resuspended in 300μl of PBS. To fix the cells, 700μl of 70% cold ethanol was added to the cell suspension while vortexing gently, and the cells were incubated on ice for 1h. After fixation, the cells were washed once with cold PBS and resuspended with 250μl of PBS, followed by PI staining with 10μl of 1mg/ml PI solution; thereafter, the cells were analyzed using flow cytometer.

### Characterization of the OsCFS Active Molecule

To characterize the properties of the anti-proliferative agent produced by *O. splanchnicus*, OsCFS and RCM (as a control) were first heated at 100°C for 10min. Next, the proteinaceous property of OsCFS was confirmed by testing whether its activity was sensitive to proteolytic enzymes. OsCFS and RCM were treated with pepsin and trypsin (10μg/ml; Sigma, MA, United States) and incubated for 1h at 37°C. To prepare the protein fraction, 500ml of OsCFS or RCM was incubated with 40% ammonium sulfate for 1h at 4°C. The precipitated crude protein was harvested by centrifugation (7,000×*g*, 1h, 4°C); the crude protein was dissolved in 10ml of 20mM sodium phosphate buffer (pH 6.8) and dialyzed twice using dialysis tubing with a 10kDa cut-off (SpectrPor^®^, Astral Scientific, NSW, AU) for 12h at 4°C. The crude protein was quantified by BCA protein assay according to the manufacturer’s instructions (Thermo Fisher Scientific, MA, United States). An anti-proliferation assay was performed with 100μg/ml crude protein. To fractionate the OsCFS into an organic phase and aqueous phase, 500ml of OsCFS or RCM was mixed with the same volume of ethyl acetate (EtOAc) for 30min at room temperature. After separation, the organic phase was harvested and dried using a rotary evaporator (EYELA, NY, United States) under reduced pressure to obtain the crude extract. The crude extract was dissolved in 1ml of methanol. The aqueous phase was processed using the same procedure as described above. The anti-proliferation assay was performed by treating the cells with medium containing 1% organic and aqueous crude extracts.

For GC/MS analysis, the aqueous phase of the OsCFS extracts was analyzed using an Agilent 7,890 B (Agilent Technologies, CA, United States) gas chromatograph coupled with an Agilent 7000C (Agilent Technologies, CA, United States) mass selective detector. Then, 1-μl aliquots of the extracts were injected into a VF-5ms column (30m×250μm i.d., 0.5-μm film thickness; Agilent Technologies, CA, United States) using injector tower G4513A (Agilent Technologies, CA, United States) in the splitless mode. The initial GC oven temperature was 70°C, and 5min after injection, the GC oven temperature was increased at 5°C/min to 320°C and held for 5min at 320°C. Helium was used as a carrier gas, and the helium flow was kept constant at a flow rate of 1.7ml/min. Detection was achieved using MS detection in electron impact mode and full-scan monitoring mode (m/z 15–800).

### Colorectal Cancer Murine Model

CT 26 cells, a murine colon cancer cell line, were harvested and suspended in PBS at a concentration of 4×10^7^ cells/ml, and 50μl of the cell suspension was injected subcutaneously (s.c.) into the flank of 7-week-old male BALB/c mice. Mice were monitored daily; tumor size (V) was monitored using calipers to measure the length and width of the tumor and determined using the following formula: Tumor size (V)=(Width)^2^×(length)×0.5.

For OsCFS treatment, OsCFS (200μl/mouse) was peritumorally injected three times per week around the tumor, beginning 4days after the initial inoculation of the cancer cells into the mice. As control group, RCM was injected. At the end of the experiment, the mice were euthanized, and the tumors were excised and weighed.

## Results

### OsCFS Inhibits the Proliferation of Colon Cancer Cells

**Figure 1:**
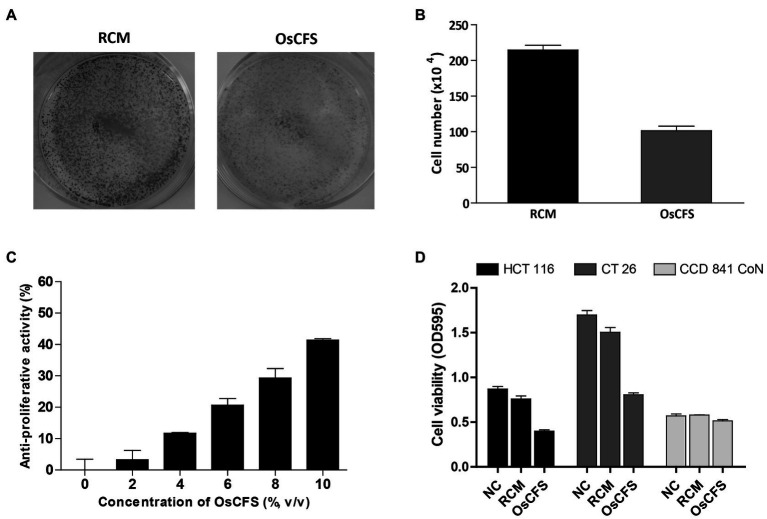
Anti-proliferative activity of the *Odoribacter splanchnicus* cell-free supernatant (OsCFS) in a cell-based system. Data are expressed as mean±standard error of three independent experiments. **(A)** The crystal violet staining assay after RCM or OsCFS treatment in HCT 116 cells. **(B)** The differences in cell number after treatment of HCT 116 cells with RCM or OsCFS for 3days. **(C)** The dose-dependent effects of OsCFS in an anti-proliferative assay. **(D)** The selective inhibiting activity of OsCFS observed in colorectal cancer cells (HCT 116, CT 26) but not in normal colon cells (CCD 841 CoN).

To efficiently screen a large amount of gut microbiota for anti-CRC activity in a high-throughput manner, we decided to use the microbiota-produced CFS, which was previously used to assess anti-CRC activity in colonic carcinoma cell lines (Chen et al., 2017; Bahmani et al., 2019). First, we sought to isolate as many different species of gut microbes as possible – other than *Lactobacillus* spp. that have mainly been studied in probiotic research – from 100 fecal samples of healthy individuals who did not take any medication. As a result of isolation, we obtained 300 different species from fecal samples. We then prepared 100 CFSs called GM01 to 100 from different liquid culturable species and examined the anti-proliferative activity of the prepared CFSs in the HCT 116 cell-based system. As shown in Supplementary Table S1, 21 CFSs out of the screened 100 samples exhibited more than 30% anti-proliferative activity against HCT 116 cells, and some of these CFSs also exhibited anti-proliferative activities against the mouse colon cancer cell line, CT 26. As we intended to perform animal experiments using the CT 26 allograft model of CRC, the CFS showing the highest activity against CT 26 cells, GM07, was chosen for further investigation for the identification of novel gut microbiota exhibiting anti-CRC activity. GM07 was identified as *O. splanchnicus* by 16S rRNA gene sequencing (99.52%, 16S rRNA gene sequence similarity) and phylogenetic analysis (Supplementary Figure S1). We confirmed that compared to RCM, OsCFS – the CFS of *O. splanchnicus* – exhibited anti-proliferative activity against HCT 116 cells, as evaluated through a crystal violet staining assay (Figure 1A) and cell counting (Figure 1B). Additionally, OsCFS inhibited the proliferation of colon cancer cells in a dose-dependent manner (Figure 1C). To evaluate whether the anti-proliferative activity of OsCFS was specific to cancer cells, OsCFS was tested on a CCD 841 CoN cell line (normal colon epithelial cells). While OsCFS exhibited 40 and 50% anti-proliferative activity on HCT 116 and CT 26 cancer cells, respectively, interestingly, no significant cytotoxic effect of OsCFS was observed on the CCD 841 CoN cell line, with less than 10% growth inhibition (Figure 1D). These results indicate that OsCFS inhibits the proliferation of colon cancer cells but not of normal colon epithelial cells.

### OsCFS Induces Apoptosis in Colon Cancer Cells

**Figure 2:**
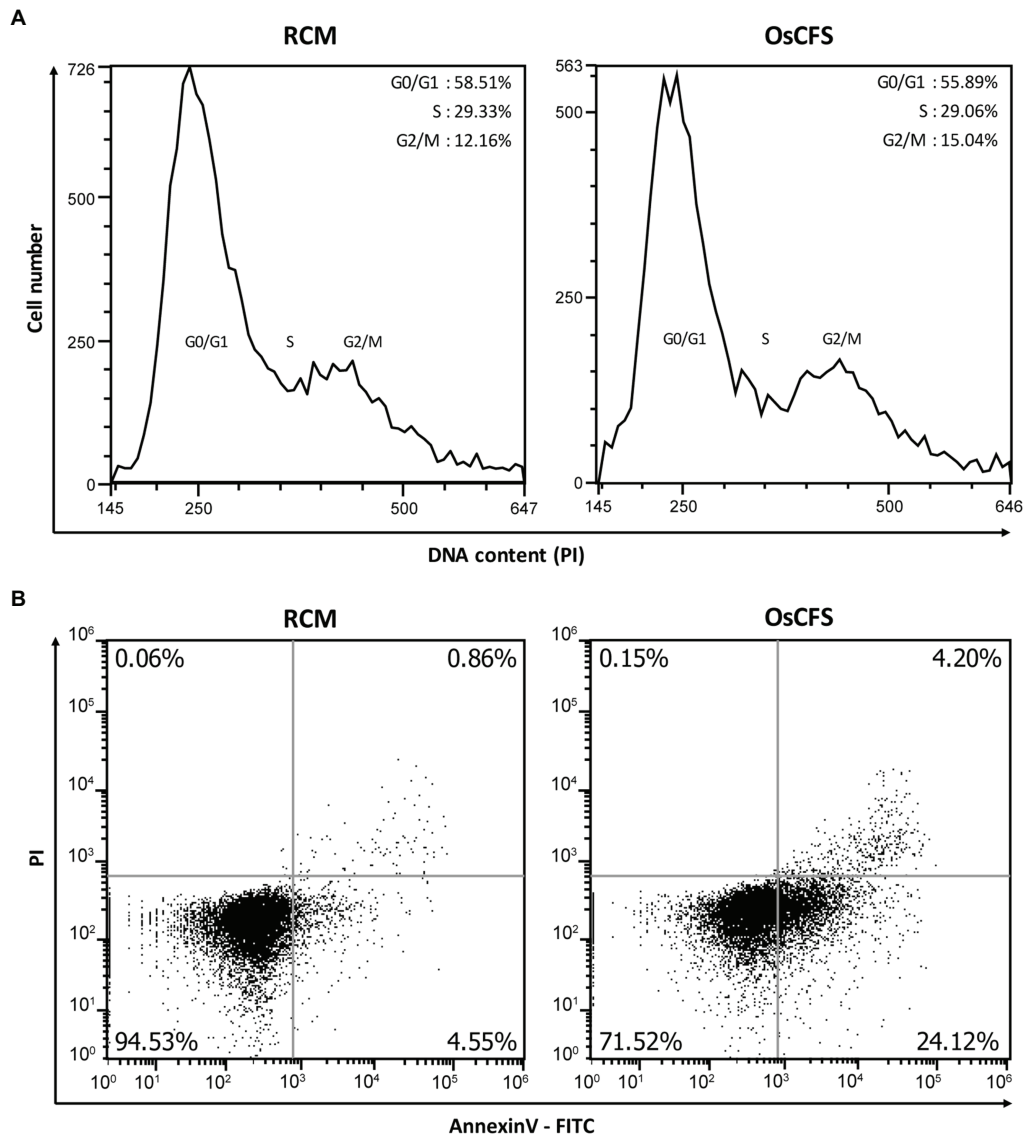
Flow cytometric analysis of HCT 116 cells treated with RCM or *Odoribacter splanchnicus* cell-free supernatant (OsCFS). **(A)** Representative histogram for cell cycle distribution using PI staining. **(B)** Representative dot plots for measurements of cell apoptosis using Annexin V-FITC and PI staining.

OsCFS exerted an anti-proliferative effect on colon cancer cell lines. To investigate whether the OsCFS-induced anti-proliferative activity was related to the cell cycle or apoptosis, both known to be closely associated with cell proliferation, we performed flow cytometric analysis (Gérard and Goldbeter, 2014). First, we determined whether OsCFS had the potential to arrest the cell cycle. The distribution of DNA content in HCT 116 cells treated with OsCFS or RCM as a control was analyzed by flow cytometry. The cell cycle DNA distribution of HCT 116 cells treated with OsCFS showed almost no change compared to that of RCM-treated cells (Figure 2A). Next, to determine whether the anti-proliferative activity of OsCFS was related to apoptosis, we evaluated apoptotic cell populations in OsCFS-treated cells by using FITC-Annexin V and PI double staining. Flow cytometric analysis showed that OsCFS-treated HCT 116 cells showed increases of 19.57 and 3.34% in the early- and late-stage apoptotic cell populations, respectively, compared to the RCM-treated cells (Figure 2B). This result suggests that OsCFS exerts its anti-proliferative effect in HCT 116 cells through the induction of apoptotic cell death in the early phase.

### OsCFS Active Molecule May Be a Metabolite and Not a Protein

**Figure 3:**
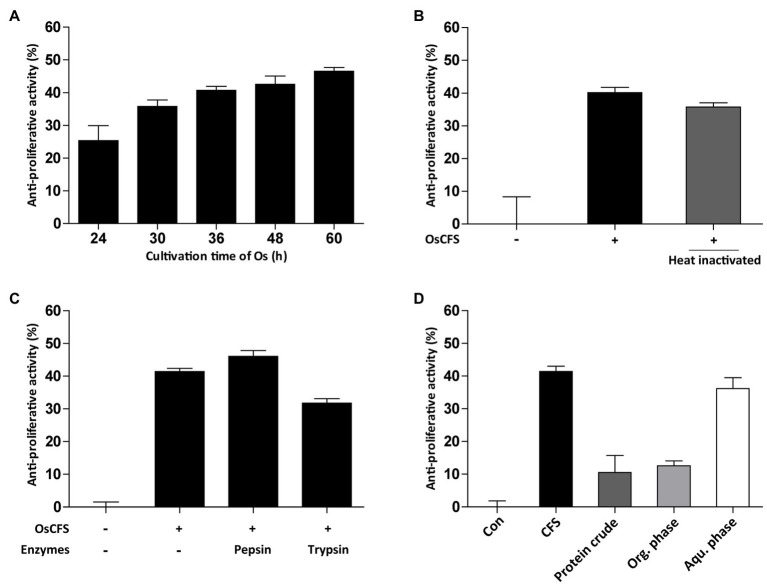
Characterization of active substances from *Odoribacter splanchnicus* cell-free supernatant (OsCFS). Data are expressed as mean±standard error of three independent experiments. **(A)** The production level of active substances depending on cultivation time of *O. splanchnicus*. **(B,C)** The effect of heat-inactivation and proteinase treatment on anti-proliferative activity. **(D)** The anti-proliferative activity of protein crude (precipitated proteins by ammonium sulfate), organic phase (Org. phase), and aqueous phase (Aqu. Phase) of the EtOAc extract.

Next, we examined the characteristics of the active molecules with anti-proliferative activity in the OsCFS, which may be a prerequisite for their identification. First, the effect of the cultivation time of *O. splanchnicus* on the anti-proliferative activity was evaluated from 24 to 60h. As shown in Figure 3A, the level of anti-proliferative activity exerted by OsCFS gradually increased over time, suggesting that the active molecules were produced and stable during cultivation. Second, to assess whether the anti-proliferative activity was influenced by heat, OsCFS was heated for 10min at 100°C. Although there was a slight decrease in the anti-proliferative activity, the heated OsCFS still exerted an anti-proliferative activity against HCT 116 cells (Figure 3B). Third, to test whether the anti-proliferative activity of OsCFS was affected by proteinase treatment, OsCFS was treated with pepsin and trypsin. As shown in Figure 3C, the proteases did not inhibit OsCFS anti-proliferative activity. Finally, we extracted the active molecules of OsCFS using EtOAc or precipitated them using ammonium sulfate. While the precipitated crude protein and the organic phase of the extracted crude extract did not show any anti-proliferative activity, the aqueous phase of the extracted crude molecules did exhibit this activity (Figure 3D). Based on these results, we assumed that the active molecules exerting anti-proliferative activity may be metabolites with properties of heat and protease stability.

### GC/MS Analysis of the OsCFS Extract

**Figure 4:**
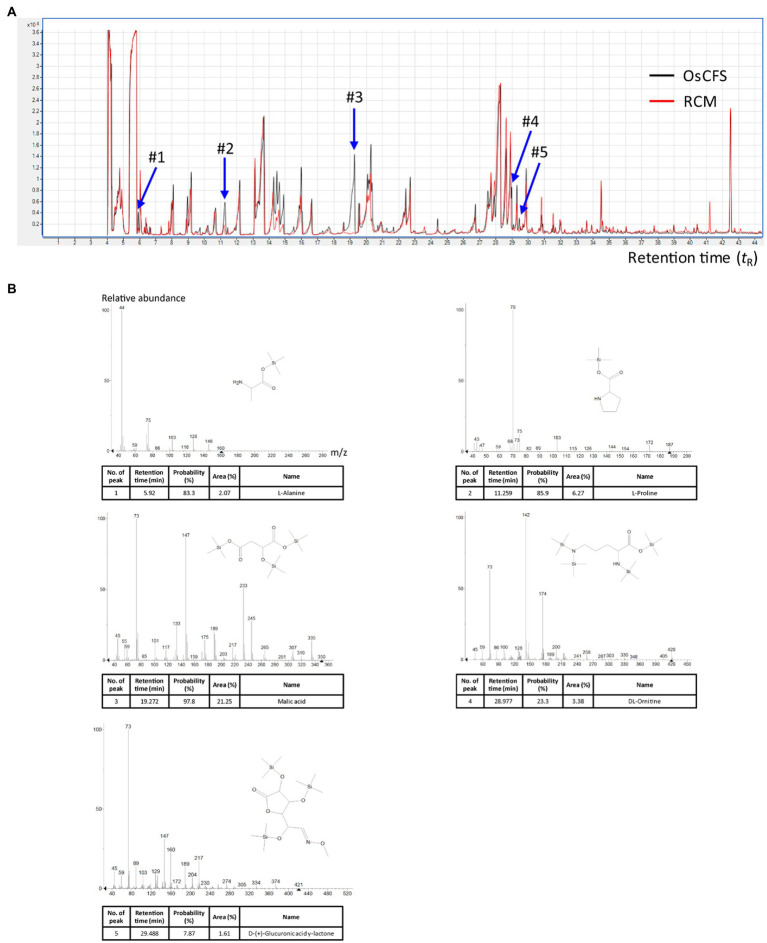
Metabolomic analysis with gas chromatography–mass spectrometry (GC–MS) to identify potential metabolites. The total ion chromatograms (TICs) of the aqueous phase of the extract were obtained by GC–MS analysis. **(A)** The merged TICs of the aqueous phase of the extracts of RCM and *Odoribacter splanchnicus* cell-free supernatant (OsCFS). The red and black peaks, respectively, indicate a representative sample from the aqueous phase of the extract of OsCFS and RCM. **(B)** Mass spectra of putative compounds from differential peaks (#1-#5) matching against mass spectral libraries (NIST).

To further investigate active compounds in the aqueous phase of the OsCFS extract, we conducted a GC/MS analysis to compare the metabolites in the extracts of RCM and OsCFS. In the total chromatogram of the GC/MS analysis, five different peaks were detected for the OsCFS extract, unlike the RCM extract (Figure 4A). Based on the mass spectra obtained from the National Institute of Standards and Technology (NIST) library, peak 1 (*t*_R_ 5.92min), peak 2 (*t*_R_ 11.26min), peak 3 (*t*_R_ 19.27min), peak 4 (*t*_R_ 28.98min), and peak 5 (*t*_R_ 29.49min) were identified as L-alanine, L-proline, malic acid, DL-ornithine, and D-(+)-glucuronic acid γ-lactone, respectively. However, DL-ornithine and D-(+)-glucuronic acid γ-lactone were detected with low probability (< 70%) as a negative match. In addition, since malic acid had the highest peak area percentage of 21.25% (whereas L-alanine and L-proline showed peak area percentages of 2.07 and 6.27%, respectively), we speculated that the anti-proliferative agent produced by *O. splanchnicus* might be malic acid (Figure 4B). Furthermore, we confirmed by HPLC analysis that a main peak of the aqueous phase of OsCFS extract was consistent with that of malic acid (Supplementary Figure S2). Additionally, malic acid exhibited anti-proliferative activity on HCT 116 cells in dose-dependent manner (Supplementary Figure S3).

### OsCFS Suppresses Tumor Growth in a Murine Allograft Model of CRC

**Figure 5:**
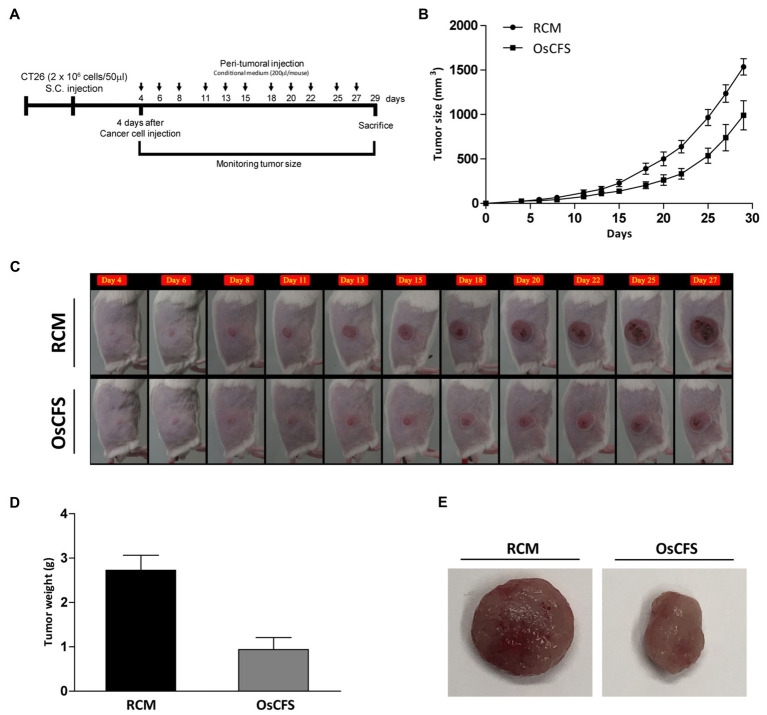
Inhibitory effect on colonic tumorigenesis of the *Odoribacter splanchnicus* cell-free supernatant (OsCFS) in a murine allograft model of CRC. Data are expressed as mean±standard error of these groups (RCM group=5, OsCFS group=5). **(A)** Schematic diagram showing the experimental design of the colorectal cancer murine model. **(B,C)** Tumor volume of mice treated with control medium or OsCFS during the experiment. **(D)** Tumor weight of mice treated with control medium or OsCFS at the end of the experiment. **(E)** Photograph of excised tumors at the end of the experiment.

We used the CT 26 allograft model of CRC to investigate the anti-CRC activity of OsCFS *in vivo*. According to the established mouse model, CT 26 mouse colon cancer cells were subcutaneously inoculated into the flanks of BALB/c mice. Four days after tumor cell injection, OsCFS or RCM (control group) was peritumorally injected every 2days until the end of the experiment. The tumor size was measured and calculated according to the tumor volume (Figure 5A). As time passed, the growth of the tumors in the OsCFS-treated group was gradually suppressed compared to that in the control group. At the end of this experiment, the tumor volume in the OsCFS-treated group was 35.6% lower than that in the control group (Figures 5B,C). To examine the tumor weight and size, CT 26 tumors were excised from euthanized mice. The tumor weight and volume were significantly reduced in OsCFS-treated mice compared to those in RCM-treated mice (Figures 5D,E). These results indicated that OsCFS exhibits anti-CRC activity *in vivo*.

## Discussion

CRC is a major disease-causing malignancies of the gastrointestinal tract, which threatens health worldwide. The pathogenesis of CRC is closely linked to the intestinal environment (Haggar and Boushey, 2009); it has been reported using metagenomic analysis that the gut microbial environment (diversity and population) in CRC patients is different from that of healthy individuals (Montalban-Arques and Scharl, 2019). The restoration of an imbalanced gut microbiota using probiotics is considered to be a potential strategy for the prevention and treatment of CRC (Ambalam et al., 2016). Moreover, in the treatment of CRC-related IBD, diverse putative beneficial bacteria, including *Clostridium*, Firmicutes spores, *Bacteroides*, *Roseburia, Prevotella*, and *Alistipes,* isolated from healthy individuals have been examined as novel biotherapeutic bacteria (Dziarski et al., 2016; Cohen et al., 2019). Although the potential of gut microbiota for CRC prevention and therapy has been reported through metagenomic analysis, little is known about the gut microbiota in relation to CRC treatment, with the exception of LAB, *Clostridium*, and *Bacillus* (Pool-Zobel et al., 1996; Chen et al., 2015). Therefore, to discover novel gut microbiota that can be potentially used for CRC treatment, we sought to identify the gut microbiota with the potential for anti-CRC activity from the gut of healthy individuals. To do this, we first isolated many different species of gut microbiota from the feces of healthy individuals, except for LAB. To screen the gut microbes for anti-CRC activity on a large scale, we prepared CFSs from the isolated gut microbiota because CFSs were previously reported to prevent the growth of cancer cells and modulate the host immune system (Escamilla et al., 2012; De Marco et al., 2018) and were the easiest material to obtain from the microbes for *in vitro* assays. However, gut microbiota have been reported to be difficult to culture (Ito et al., 2019) as only 100 out of the 300 species of gut microbiota can be cultured in RCM liquid medium. To discover other gut microbiota related to CRC treatment, further experiments using different media and culture conditions will be needed to study the gut microbiota that could not be cultured in this study. In addition, cell extracts, cell lysates, dead or live bacteria, and bacteria-secreted molecules in response to a host stimulus can be employed as alternative materials for anti-CRC activity screening, as has been done in previous studies to treat CRC *in vitro* or *in vivo* (Lee et al., 2008; Cipriani et al., 2011; Levy et al., 2015; Chung et al., 2019; Zhuo et al., 2019). Moreover, the CFSs of 20 gut microbes that showed anti-proliferative activity against HCT 116 cells but low activity against CT 26 cells can be further examined for anti-CRC activity using the HCT 116 xenograft mouse model. Thus, more candidates with anti-CRC activity from our isolated gut microbiota may be identified in future studies.

Because cancer cells are highly proliferative, anti-proliferative activity has been investigated as one of the major targets in the development of anti-cancer drugs (Dembic, 2020). For instance, the anti-proliferative activities of traditional herbal and marine derivative products were screened to assess their potential anti-cancer effects and consequently develop anti-cancer drugs (da Silva et al., 2019; Cui et al., 2020). Therefore, to identify anti-CRC candidates from the gut microbiota, we examined the anti-proliferative activity of these candidates against HCT 116 cells *in vitro*. Our results showed that CFSs of 21 candidates from the 100 screened gut microbiota exerted anti-proliferative activity on HCT 116 cells. In addition, prior to determining the anti-CRC activity of the candidates in the CT 26 allograft model of CRC (a widely used animal model for cancer drug development that allows for quick and cost-effective experimental testing of potential drugs), the anti-proliferative activity of the CFSs of 21 candidates was examined against CT 26 cells *in vitro*. Previous reports have shown that the level of anti-proliferative activity differs depending on the cell line (Savic et al., 2020; Shin et al., 2021); thus, some of the CFSs exerted no or little anti-proliferative activity against CT 26 cells. Of the 21 candidates, the CFS of GM07 exerted the highest anti-proliferative effect against CT 26 cells; hence, GM07 was chosen for further investigation and the generative gut microbe was identified as *O. splanchnicus* through phylogenetic analysis.

Cell proliferation is closely related to apoptosis and the regulation of the cell cycle (Alenzi, 2004). Hence, there are many anti-cancer drugs that target the stimulation of apoptosis or the regulation of the cell cycle (Fulda and Debatin, 2006). In this study, we examined whether OsCFS induced apoptosis or cell cycle arrest *in vitro* by using flow cytometry. Our data indicated that OsCFS inhibited the proliferation of HCT 116 cells through the induction of apoptotic cell death in the early phase but not through cell cycle arrest. A recent study demonstrated that the extract of *Lactobacillus plantarum* strain 06CC2 induced mitochondrial-mediated apoptosis through the activation of c-Jun N-terminal kinase (JNK)/p38 mitogen-activated protein kinase (p38 MAPK) signaling in CRC cells (Hiraishi et al., 2019). In addition, *Bifidobacterium* sp., which has a significant anti-CRC effect *in vitro* and *in vivo*, markedly induced apoptosis in the early phase through the downregulation of the expression of epidermal growth factor receptor (EGFR) and human epidermal growth factor receptor 2 (HER-2), both of which are related to cell proliferation (Parisa et al., 2020). The mechanism of OsCFS-induced apoptosis could be the same as those mentioned above or different; this needs to be further studied in the future.

*Odoribacter splanchnicus* is a commensal bacterium that inhabits the human intestinal tract (Werner et al., 1975). Previous reports have indicated that *O. splanchnicus* may beneficially activate the intestinal health. First, Werner et al. demonstrated that *O. splanchnicus* could produce various SCFAs, including acetic acid, propionic acid, succinic acid, butyric acid, isovaleric acid, and isobutyric acid. Some studies have indicated that butyric acid induces autophagy, thereby inhibiting the proliferation of CRC cells (Donohoe et al., 2011; Lee et al., 2012; Lee and Lee, 2012). Second, Hiippala et al. demonstrated that OMVs produced by *O. splanchnicus* exert immunoregulatory effects by reducing IL-8 production in lipopolysaccharide (LPS)-stimulated HT-29 cells and inducing IL-10 production (Hiippala et al., 2020). In addition, bacterial OMVs have been demonstrated to be potent anti-cancer agents by effecting a significant reduction in tumor growth *in vivo* (Qing et al., 2020). Third, *O. splanchnicus* lacks the *lpxM* gene needed for endotoxic hexa-acylated LPS and instead harbors penta-acylated LPS, which is 100-fold less toxic than *E. coli* LPS (Han et al., 2013). The non-toxic penta-acylated LPS silences toll-like receptor 4 (TLR4) signaling, leading to lower cytokine release through the TLR4-TRAM-TRIF pathway (Park et al., 2009; Han et al., 2013). Finally, in case of human physiopathology, the abundance of *Odoribacter* spp. was reduced in the patients with IBD or CRC as mentioned above. Moreover, in metastatic renal cell carcinoma, most abundant species among patients with clinical benefit were *Bifidobacterium adolescentis*, *Barnesiella intestinihominis*, *O. splanchnicus*, and *Bacteroides eggerthii* (Salgia et al., 2020). Accordingly, the above studies suggest that *O. splanchnicus* may have an anti-CRC effect as beneficial probiotics. In this study, we clearly demonstrated for the first time that CFS of *O. splanchnicus* had anti-CRC activity through anti-proliferative molecules *in vitro* and *in vivo*.

Although anti-proliferative agents in the OsCFS have not yet been completely identified, the results of our preliminary experiments showed that the bioactive molecules in the OsCFS are heat-stable and non-proteinaceous. In addition, the GC/MS analysis suggested that the putative anti-proliferative molecule in the aqueous phase of OsCFS might be malic acid. Malic acid is an organic compound that has been reported to have many bioactive functions, such as antioxidant activity, capturing free radicals, and antimicrobial activity against some pathogenic bacteria (Eswaranandam et al., 2006; Zhang et al., 2020). In addition, malic acid can be chemically polymerized to polymalic acid, which has a wide range of applications in cancer therapy because of its biochemical properties, including its biocompatibility, biodegradability, and chemical modifiability (Zeng et al., 2019). Moreover, this study showed that malic acid exerted the anti-proliferative activity *in vitro*. However, it has been still not conclusive that malic acid is indeed the active agent for the anti-CRC activity of OsCFS because this study did not show whether *O. splanchnicus* can produce malic acid *in vivo* and active agents other than malic acid may still be responsible for the anti-CRC activity of OsCFS, which should also be further studied.

In the present study, we evaluated whether OsCFS was associated with the suppression of CRC development in CT 26 tumor-bearing mice. Our results indicate that the peritumoral injection of OsCFS significantly reduced CRC tumorigenesis in a mouse allograft model. Anti-CRC tumorigenesis can also be exerted by the direct interactions between beneficial bacteria and intestinal mucosa or other gut microflora, and previous studies showed that oral ingestion of probiotics enhanced the intestinal mucosal barrier (Costello et al., 2014; Liu et al., 2016) and improved the density and diversity of mucosa-associated microbiota in patients with CRC (Gao et al., 2015). However, administration of live *O. splanchnicus* by oral gavage did not show an inhibitory effect on tumor growth (data not shown). According to recent report, the mucosal adherent ability of *O. splanchnicus* was found to be below the 1% background binding level and *O. splanchnicus* is thus considered to be non-adherent to epithelial cells (Hiippala et al., 2020). Therefore, we speculate that *O. splanchnicus* did not inhibit tumor growth in an orally administered mouse model possibly due to its low adherence or non-production of active molecules as CFS in the gastrointestinal tract. Further study to improve the ability of *O. splanchnicus* colonization to host intestine and the productivity of its anti-CRC agents *in vivo* would be required.

Although the metagenomic analysis demonstrated that CRC was closely associated with gut microbiota, there is little direct evidence for this association obtained using isolated gut microbes, except LAB. In this study, we clearly showed that the CFS of *O. splanchnicus* inhibited the proliferation of CRC cells and ameliorated tumorigenesis in a mouse allograft model of CRC. Accordingly, our study provides a useful information for the development of potential novel probiotics for the prevention and treatment of CRC.

## Data Availability Statement

The datasets presented in this study can be found in online repositories. The names of the repository/repositories and accession number(s) can be found at: https://www.ncbi.nlm.nih.gov/genbank/, MW325948.

## Ethics Statement

The animal study was reviewed and approved by Institutional Animal Care and Use Committee of the Korea Research Institute of Bioscience and Biotechnology (approval number: KRIBB-AEC-18063).

## Author Contributions

BO, WC, and JL: conceptualization. BO and WC: data curation. J-SL and JL: funding acquisition. BO, WC, J-SK, SR, SY, S-HP, SK, JL, WJ, Y-MK, J-HJ, and JL: methodology. BO and WC: software. BO, WC, J-SK, SR, SY, S-HP, SK, JL, WJ, Y-MK, J-HJ, and JL: investigation. JL: resources. BO: writing - original draft. JL: writing-review and editing. All authors contributed to the article and approved the submitted version.

## Funding

This work was supported by the Bio & Medical Technology Development Program of the National Research Foundation of Korea (NRF) funded by the Ministry of Science (NRF-2016M3A9F3947962 and NRF-2019M3A9F3065226) and ICT (MSIT) of the Republic of Korea, and a grant from the Korea Research Institute of Bioscience & Biotechnology (KRIBB) Research Initiative Program.

## Conflict of Interest

The authors declare that the research was conducted in the absence of any commercial or financial relationships that could be construed as a potential conflict of interest.

## Publisher’s Note

All claims expressed in this article are solely those of the authors and do not necessarily represent those of their affiliated organizations, or those of the publisher, the editors and the reviewers. Any product that may be evaluated in this article, or claim that may be made by its manufacturer, is not guaranteed or endorsed by the publisher.

## Footnotes

^1^ http://www.ezbiocloud.net

## Abbreviations

CRC: colorectal cancer
CFS: cell-free supernatant
EtOAc: ethyl acetate
IBD: inflammatory bowel disease
LAB: lactic acid bacteria
LPS: lipopolysaccharide
Os: *Odoribacter splanchnicus*
SCFA: short-chain fatty acid
RCM: reinforced clostridial medium

## References

[ref1] Alenzi F. Q. (2004). Links between apoptosis, proliferation and the cell cycle. Br. J. Biomed. Sci. 61, 99–102. doi: 10.1080/09674845.2004.11732652, PMID: 15250676

[ref2] Ambalam P. Raman M. Purama R. K. Doble M. (2016). Probiotics, prebiotics and colorectal cancer prevention. Best Pract. Res. Clin. Gastroenterol. 30, 119–131. doi: 10.1016/j.bpg.2016.02.009, PMID: 27048903

[ref3] Bahmani S. Azarpira N. Moazamian E. (2019). Anti-colon cancer activity of Bifidobacterium metabolites on colon cancer cell line SW742. Turk. J. Gastroenterol. 30, 835–842. doi: 10.5152/tjg.2019.18451, PMID: 31530527 PMC6750814

[ref4] Chen Z. F. Ai L. Y. Wang J. L. Ren L. L. Yu Y. N. Xu J.. (2015). Probiotics clostridium butyricum and Bacillus subtilis ameliorate intestinal tumorigenesis. Future Microbiol. 10, 1433–1445. doi: 10.2217/fmb.15.66, PMID: 26346930

[ref5] Chen Z. Y. Hsieh Y. M. Huang C. C. Tsai C. C. (2017). Inhibitory effects of probiotic lactobacillus on the growth of human colonic carcinoma cell line HT-29. Molecules 22:107. doi: 10.3390/molecules22010107 PMC6155858 28075415

[ref6] Chung I. C. Ouyang C.-N. Yuan S.-N. Lin H.-C. Huang K.-Y. Wu P.-S.. (2019). Pretreatment with a heat-killed probiotic modulates the NLRP3 inflammasome and attenuates colitis-associated colorectal cancer in mice. Nutrients 11:516. doi: 10.3390/nu11030516, PMID: 30823406 PMC6471765

[ref7] Cipriani S. Mencarelli A. Chini M. G. Distrutti E. Renga B. Bifulco G.. (2011). The bile acid receptor GPBAR-1 (TGR5) modulates integrity of intestinal barrier and immune response to experimental colitis. PLoS One 6:e25637. doi: 10.1371/journal.pone.0025637, PMID: 22046243 PMC3203117

[ref8] Cohen L. J. Cho J. H. Gevers D. Chu H. (2019). Genetic factors and the intestinal microbiome guide development of microbe-based therapies for inflammatory bowel diseases. Gastroenterology 156, 2174–2189. doi: 10.1053/j.gastro.2019.03.017, PMID: 30880022 PMC6568267

[ref9] Costello C. M. Sorna R. M. Goh Y.-L. Cengic I. Jain N. K. March J. C. (2014). 3-D intestinal scaffolds for evaluating the therapeutic potential of probiotics. Mol. Pharm. 11, 2030–2039. doi: 10.1021/mp5001422, PMID: 24798584 PMC4096232

[ref10] Cui H. Bashar M. A. E. Rady I. El-Naggar H. A. Abd El-Maoula L. M. Mehany A. B. M. (2020). Antiproliferative activity, proapoptotic effect, and cell cycle arrest in human cancer cells of some marine natural product extract. Oxidative Med. Cell. Longev. 2020, 1–12. doi: 10.1155/2020/7948705 PMC7714591 33294124

[ref11] Da Silva A. C. N. Do Nascimento R. M. C. Rodrigues D. C. D. N. Ferreira P. M. P. Pessoa C. Lima D. J. B.. (2019). In vitro activity evaluation of seven Brazilian Asteraceae against cancer cells and Leishmania amazonensis. S. Afr. J. Bot. 121, 267–273. doi: 10.1016/j.sajb.2018.11.008

[ref12] De Marco S. Sichetti M. Muradyan D. Piccioni M. Traina G. Pagiotti R.. (2018). Probiotic cell-free supernatants exhibited anti-inflammatory and antioxidant activity on human gut epithelial cells and macrophages stimulated with LPS. Evid. Based Complement. Alternat. Med. 2018, 1–1756308, 12. doi: 10.1155/2018/1756308 PMC6057331 30069221

[ref13] Dembic Z. (2020). Antitumor drugs and their targets. Molecules 25:5776. doi: 10.3390/molecules25235776, PMID: 33297561 PMC7730053

[ref14] Donohoe D. R. Garge N. Zhang X. Sun W. O'connell T. M. Bunger M. K.. (2011). The microbiome and butyrate regulate energy metabolism and autophagy in the mammalian colon. Cell Metab. 13, 517–526. doi: 10.1016/j.cmet.2011.02.018, PMID: 21531334 PMC3099420

[ref15] Dziarski R. Park S. Y. Kashyap D. R. Dowd S. E. Gupta D. (2016). Pglyrp-regulated gut microflora Prevotella falsenii, Parabacteroides distasonis and Bacteroides eggerthii enhance and Alistipes finegoldii attenuates colitis in mice. PLoS One 11:e0146162. doi: 10.1371/journal.pone.0146162, PMID: 26727498 PMC4699708

[ref16] Escamilla J. Lane M. A. Maitin V. (2012). Cell-free supernatants from probiotic lactobacillus casei and lactobacillus rhamnosus GG decrease colon cancer cell invasion in vitro. Nutr. Cancer 64, 871–878. doi: 10.1080/01635581.2012.700758, PMID: 22830611

[ref17] Eswaranandam S. Hettiarachchy N. Johnson M. (2006). Antimicrobial activity of citric, lactic, malic, or tartaric acids and Nisin-incorporated soy protein film against listeria monocytogenes, *Escherichia coli* O157:H7, and salmonella gaminara. J. Food Sci. 69, FMS79–FMS84. doi: 10.1111/j.1365-2621.2004.tb13375.x

[ref18] Fang C.-Y. Chen J.-S. Hsu B.-M. Hussain B. Rathod J. Lee K.-H. (2021). Colorectal cancer stage-specific fecal bacterial community fingerprinting of the Taiwanese population and underpinning of potential taxonomic biomarkers. Microorganisms 9:1548. doi: 10.3390/microorganisms9081548, PMID: 34442626 PMC8401100

[ref19] Ferreira-Halder C. V. Faria A. V. D. S. Andrade S. S. (2017). Action and function of Faecalibacterium prausnitzii in health and disease. Best Pract. Res. Clin. Gastroenterol. 31, 643–648. doi: 10.1016/j.bpg.2017.09.011, PMID: 29566907

[ref20] Fulda S. Debatin K. M. (2006). Extrinsic versus intrinsic apoptosis pathways in anticancer chemotherapy. Oncogene 25, 4798–4811. doi: 10.1038/sj.onc.1209608, PMID: 16892092

[ref21] Gagnière J. Raisch J. Veziant J. Barnich N. Bonnet R. Buc E.. (2016). Gut microbiota imbalance and colorectal cancer. World J. Gastroenterol. 22, 501–518. doi: 10.3748/wjg.v22.i2.501, PMID: 26811603 PMC4716055

[ref22] Galdeano C. M. Perdigón G. (2006). The probiotic bacterium lactobacillus casei induces activation of the gut mucosal immune system through innate immunity. Clin. Vaccine Immunol. 13, 219–226. doi: 10.1128/CVI.13.2.219-226.2006, PMID: 16467329 PMC1391937

[ref23] Gao Z. Guo B. Gao R. Zhu Q. Wu W. Qin H. (2015). Probiotics modify human intestinal mucosa-associated microbiota in patients with colorectal cancer. Mol. Med. Rep. 12, 6119–6127. doi: 10.3892/mmr.2015.4124, PMID: 26238090

[ref24] Gérard C. Goldbeter A. (2014). The balance between cell cycle arrest and cell proliferation: control by the extracellular matrix and by contact inhibition. Interface Focus 4, 20130075–20130075. doi: 10.1098/rsfs.2013.0075, PMID: 24904738 PMC3996587

[ref25] Haggar F. A. Boushey R. P. (2009). Colorectal cancer epidemiology: incidence, mortality, survival, and risk factors. Clin. Colon Rectal Surg. 22, 191–197. doi: 10.1055/s-0029-1242458, PMID: 21037809 PMC2796096

[ref26] Han Y. Li Y. Chen J. Tan Y. Guan F. Wang X. (2013). Construction of monophosphoryl lipid A producing Escherichia coli mutants and comparison of immuno-stimulatory activities of their lipopolysaccharides. Mar. Drugs 11, 363–376. doi: 10.3390/md11020363, PMID: 23434832 PMC3640385

[ref27] Hiippala K. Barreto G. Burrello C. Diaz-Basabe A. Suutarinen M. Kainulainen V.. (2020). Novel *Odoribacter splanchnicus* strain and its outer membrane vesicles exert immunoregulatory effects in vitro. Front. Microbiol. 11:2906. doi: 10.3389/fmicb.2020.575455 PMC7689251 33281770

[ref28] Hinnebusch B. F. Meng S. Wu J. T. Archer S. Y. Hodin R. A. (2002). The effects of short-chain fatty acids on human colon cancer cell phenotype are associated with histone hyperacetylation. J. Nutr. 132, 1012–1017. doi: 10.1093/jn/132.5.1012, PMID: 11983830

[ref29] Hiraishi N. Kanmura S. Oda K. Arima S. Kumagai K. Mawatari S.. (2019). Extract of *Lactobacillus plantarum* strain 06CC2 induces JNK/p38 MAPK pathway-mediated apoptosis through endoplasmic reticulum stress in Caco2 colorectal cancer cells. Biochemi. Biophys. Rep. 20, 100691. doi: 10.1016/j.bbrep.2019.100691, PMID: 31650040 PMC6804738

[ref30] Ito T. Sekizuka T. Kishi N. Yamashita A. Kuroda M. (2019). Conventional culture methods with commercially available media unveil the presence of novel culturable bacteria. Gut Microbes 10, 77–91. doi: 10.1080/19490976.2018.1491265, PMID: 30118379 PMC6363062

[ref31] Jemal A. Siegel R. Xu J. Ward E. (2010). Cancer statistics, 2010. CA Cancer J. Clin. 60, 277–300. doi: 10.3322/caac.20073, PMID: 20610543

[ref32] Joossens M. Huys G. Cnockaert M. De Preter V. Verbeke K. Rutgeerts P.. (2011). Dysbiosis of the faecal microbiota in patients with Crohn’s disease and their unaffected relatives. Gut 60, 631–637. doi: 10.1136/gut.2010.223263, PMID: 21209126

[ref33] Khor B. Gardet A. Xavier R. J. (2011). Genetics and pathogenesis of inflammatory bowel disease. Nature 474, 307–317. doi: 10.1038/nature10209, PMID: 21677747 PMC3204665

[ref34] Konishi H. Fujiya M. Tanaka H. Ueno N. Moriichi K. Sasajima J.. (2016). Probiotic-derived ferrichrome inhibits colon cancer progression via JNK-mediated apoptosis. Nat. Commun. 7, 12365–12365. doi: 10.1038/ncomms12365, PMID: 27507542 PMC4987524

[ref35] Kulaylat M. N. Dayton M. T. (2010). Ulcerative colitis and cancer. J. Surg. Oncol. 101, 706–712. doi: 10.1002/jso.21505, PMID: 20512947

[ref36] Lawrence G. W. Begley M. Cotter P. D. Guinane C. M. (2020). Potential use of biotherapeutic bacteria to target colorectal cancer-associated taxa. Int. J. Mol. Sci. 21, 924. doi: 10.3390/ijms21030924, PMID: 32019270 PMC7037558

[ref37] Lee D. K. Jang S. Kim M. J. Kim J. H. Chung M. J. Kim K. J.. (2008). Anti-proliferative effects of Bifidobacterium adolescentis SPM0212 extract on human colon cancer cell lines. BMC Cancer 8, 310. doi: 10.1186/1471-2407-8-310, PMID: 18950540 PMC2605768

[ref38] Lee J. S. Kim Y. J. Kim C. L. Lee G. M. (2012). Differential induction of autophagy in caspase-3/7 down-regulating and Bcl-2 overexpressing recombinant CHO cells subjected to sodium butyrate treatment. J. Biotechnol. 161, 34–41. doi: 10.1016/j.jbiotec.2012.05.011, PMID: 22728390

[ref39] Lee J. S. Lee G. M. (2012). Effect of sodium butyrate on autophagy and apoptosis in Chinese hamster ovary cells. Biotechnol. Prog. 28, 349–357. doi: 10.1002/btpr.1512, PMID: 22492702

[ref40] Levy M. Thaiss C. A. Zeevi D. Dohnalová L. Zilberman-Schapira G. Mahdi J. A.. (2015). Microbiota-modulated metabolites shape the intestinal microenvironment by regulating NLRP6 Inflammasome signaling. Cell 163, 1428–1443. doi: 10.1016/j.cell.2015.10.048, PMID: 26638072 PMC5665753

[ref41] Liu D. Jiang X.-Y. Zhou L.-S. Song J.-H. Zhang X. (2016). Effects of probiotics on intestinal mucosa barrier in patients with colorectal cancer after operation: meta-analysis of randomized controlled trials. Medicine 95, e3342–e3342. doi: 10.1097/MD.0000000000003342, PMID: 27082589 PMC4839833

[ref42] Liu W. Zhang R. Shu R. Yu J. Li H. Long H.. (2020). Study of the relationship between microbiome and colorectal cancer susceptibility using 16SrRNA sequencing. Biomed. Res. Int. 2020, 1–17. doi: 10.1155/2020/7828392 PMC7011317 32083132

[ref43] Ma E. L. Choi Y. J. Choi J. Pothoulakis C. Rhee S. H. Im E. (2010). The anticancer effect of probiotic bacillus polyfermenticus on human colon cancer cells is mediated through ErbB2 and ErbB3 inhibition. Int. J. Cancer 127, 780–790. doi: 10.1002/ijc.25011, PMID: 19876926 PMC4420487

[ref44] Marshall J. R. (2008). Prevention of colorectal cancer: diet, chemoprevention, and lifestyle. Gastroenterol. Clin. N. Am. 37, 73–82. doi: 10.1016/j.gtc.2007.12.008, PMID: 18313540 PMC2637789

[ref45] Montalban-Arques A. Scharl M. (2019). Intestinal microbiota and colorectal carcinoma: implications for pathogenesis, diagnosis, and therapy. EBioMedicine 48, 648–655. doi: 10.1016/j.ebiom.2019.09.050, PMID: 31631043 PMC6838386

[ref46] Morgan X. C. Tickle T. L. Sokol H. Gevers D. Devaney K. L. Ward D. V.. (2012). Dysfunction of the intestinal microbiome in inflammatory bowel disease and treatment. Genome Biol. 13, R79–R79. doi: 10.1186/gb-2012-13-9-r79, PMID: 23013615 PMC3506950

[ref47] Nakatsu G. Li X. Zhou H. Sheng J. Wong S. H. Wu W. K. K.. (2015). Gut mucosal microbiome across stages of colorectal carcinogenesis. Nat. Commun. 6, 8727–8727. doi: 10.1038/ncomms9727, PMID: 26515465 PMC4640069

[ref48] Orlando A. Linsalata M. Russo F. (2016). Antiproliferative effects on colon adenocarcinoma cells induced by co-administration of vitamin K1 and lactobacillus rhamnosus GG. Int. J. Oncol. 48, 2629–2638. doi: 10.3892/ijo.2016.3463, PMID: 27035094

[ref49] Parisa A. Roya G. Mahdi R. Shabnam R. Maryam E. Malihe T. (2020). Anti-cancer effects of Bifidobacterium species in colon cancer cells and a mouse model of carcinogenesis. PLoS One 15:e0232930. doi: 10.1371/journal.pone.0232930, PMID: 32401801 PMC7219778

[ref50] Park B. S. Song D. H. Kim H. M. Choi B. S. Lee H. Lee J. O. (2009). The structural basis of lipopolysaccharide recognition by the TLR4-MD-2 complex. Nature 458, 1191–1195. doi: 10.1038/nature07830, PMID: 19252480

[ref51] Pool-Zobel B. L. Neudecker C. Domizlaff I. Ji S. Schillinger U. Rumney C.. (1996). Lactobacillus- and bifidobacterium-mediated antigenotoxicity in the colon of rats. Nutr. Cancer 26, 365–380. doi: 10.1080/01635589609514492, PMID: 8910918

[ref52] Qing S. Lyu C. Zhu L. Pan C. Wang S. Li F.. (2020). Biomineralized bacterial outer membrane vesicles potentiate safe and efficient tumor microenvironment reprogramming for anticancer therapy. Adv. Mater. 32:e2002085. doi: 10.1002/adma.202002085 33015871

[ref53] Rafter J. Bennett M. Caderni G. Clune Y. Hughes R. Karlsson P. C.. (2007). Dietary synbiotics reduce cancer risk factors in polypectomized and colon cancer patients. Am. J. Clin. Nutr. 85, 488–496. doi: 10.1093/ajcn/85.2.488, PMID: 17284748

[ref54] Rowland I. R. Rumney C. J. Coutts J. T. Lievense L. C. (1998). Effect of Bifidobacterium longum and inulin on gut bacterial metabolism and carcinogen-induced aberrant crypt foci in rats. Carcinogenesis 19, 281–285. doi: 10.1093/carcin/19.2.281, PMID: 9498277

[ref55] Salgia N. J. Bergerot P. G. Maia M. C. Dizman N. Hsu J. Gillece J. D.. (2020). Stool microbiome profiling of patients with metastatic renal cell carcinoma receiving anti-PD-1 immune checkpoint inhibitors. Eur. Urol. 78, 498–502. doi: 10.1016/j.eururo.2020.07.011, PMID: 32828600

[ref56] Sánchez-Alcoholado L. Ramos-Molina B. Otero A. Laborda-Illanes A. Ordóñez R. Medina J. A.. (2020). The role of the gut microbiome in colorectal cancer development and therapy response. Cancers 12, 1406. doi: 10.3390/cancers12061406, PMID: 32486066 PMC7352899

[ref57] Savic M. Arsenijevic A. Milovanovic J. Stojanovic B. Stankovic V. Rilak Simovic A.. (2020). Antitumor activity of ruthenium(II) terpyridine complexes towards colon cancer cells in vitro and in vivo. Molecules 25:4699. doi: 10.3390/molecules25204699, PMID: 33066568 PMC7587369

[ref58] Shin M. K. Jeon Y. D. Hong S. H. Kang S. H. Kee J. Y. Jin J. S. (2021). In vivo and In vitro effects of Tracheloside on colorectal cancer cell proliferation and metastasis. Antioxidants 10:513. doi: 10.3390/antiox10040513 33806109 PMC8064450

[ref59] Vinderola G. Perdigón G. Duarte J. Farnworth E. Matar C. (2006). Effects of the oral administration of the exopolysaccharide produced by lactobacillus kefiranofaciens on the gut mucosal immunity. Cytokine 36, 254–260. doi: 10.1016/j.cyto.2007.01.003, PMID: 17363262

[ref60] Werner H. Rintelen G. Kunstek-Santos H. (1975). A new butyric acid-producing bacteroides species: B. splanchnicus n. sp.(author’s transl). Zentralblatt fur Bakteriologie, Parasitenkunde, Infektionskrankheiten und hygiene. Erste Abteilung Originale. Reihe A: Medizinische Mikrobiologie und Parasitologie 231, 133–144. 168701

[ref61] Yachida S. Mizutani S. Shiroma H. Shiba S. Nakajima T. Sakamoto T.. (2019). Metagenomic and metabolomic analyses reveal distinct stage-specific phenotypes of the gut microbiota in colorectal cancer. Nat. Med. 25, 968–976. doi: 10.1038/s41591-019-0458-7, PMID: 31171880

[ref62] Yoon S. H. Ha S. M. Kwon S. Lim J. Kim Y. Seo H.. (2017). Introducing EzBioCloud: a taxonomically united database of 16S rRNA gene sequences and whole-genome assemblies. Int. J. Syst. Evol. Microbiol. 67, 1613–1617. doi: 10.1099/ijsem.0.001755, PMID: 28005526 PMC5563544

[ref63] Zackular J. P. Baxter N. T. Iverson K. D. Sadler W. D. Petrosino J. F. Chen G. Y.. (2013). The gut microbiome modulates colon tumorigenesis. mBio 4:e00692-00613. doi: 10.1128/mBio.00692-13 24194538 PMC3892781

[ref64] Zeng W. Zhang B. Liu Q. Chen G. Liang Z. (2019). Analysis of the L-malate biosynthesis pathway involved in poly(β-L-malic acid) production in Aureobasidium melanogenum GXZ-6 by addition of metabolic intermediates and inhibitors. J. Microbiol. 57, 281–287. doi: 10.1007/s12275-019-8424-0 30721461

[ref65] Zhang L. Zhang P. Xia C. Cheng Y. Guo X. Li Y. (2020). Effects of malic acid and citric acid on growth performance, antioxidant capacity, haematology and immune response of Carassius auratus gibelio. Aquac. Res. 51, 2766–2776. doi: 10.1111/are.14616

[ref66] Zhuo Q. Yu B. Zhou J. Zhang J. Zhang R. Xie J.. (2019). Lysates of lactobacillus acidophilus combined with CTLA-4-blocking antibodies enhance antitumor immunity in a mouse colon cancer model. Sci. Rep. 9, 20128. doi: 10.1038/s41598-019-56661-y, PMID: 31882868 PMC6934597

[ref67] Zou S. Fang L. Lee M.-H. (2018). Dysbiosis of gut microbiota in promoting the development of colorectal cancer. Gastroenterol. Rep. 6, 1–12. doi: 10.1093/gastro/gox031 PMC5806407 29479437

