# Supplementary material for: Cell-Free Supernatant of Odoribacter splanchnicus Isolated From Human Feces Exhibits Anti-colorectal Cancer Activity
Source: Front Microbiol. 2021 Nov 11;12:736343. doi: 10.3389/fmicb.2021.736343 (PMC8638082; doi:10.3389/fmicb.2021.736343)
Supplement: Supplementary file 1 [file Data_Sheet_1.pdf]

Frontiers in Microbiology

Cell-Free Supernatant of *Odoribacter splanchnicus* Isolated From Human Feces Exhibits Anti-colorectal Cancer Activity

Byeong Seob Oh[1†], Won Jung Choi[1†], Ji-Sun Kim[1], Seoung Woo Ryu[1], Seung Yeob Yu[1], Jung-Sook Lee[1], Seung-Hwan Park[1], Se Won Kang[1], Won Yong Jung[2], Young-Min Kim[3], Jae-Ho Jeong[4] and Ju Huck Lee[1*]

[1]Korean Collection for Type Cultures, Biological Resource Center, Korea Research Institute of Bioscience and Biotechnology, Jeongeup 56212, Republic of Korea.

[2]Korean Bioinformation Center, Korea Research Institute of Bioscience and Biotechnology, Daejeon 34141, Republic of Korea.

[3]Department of Food Science and Technology, and Bio-energy Research Center, Chonnam National University, Gwangju 61186, Republic of Korea.

[4]Department of Microbiology, Chonnam National University Medical School, Gwangju, Republic of Korea.

Running header: *O. splanchnicus* Exhibits Anti-colorectal Cancer Activity

Keywords: gut microbiota, *Odoribacter splanchnicus*, colorectal cancer, cell-free supernatant, apoptosis and murine model

***Corresponding authors**
Ju Huck Lee, Ph.D. Tel: +82-63-570-5634, Fax: +82-63-570-5609**,** E-mail: juhuck@kribb.re.kr

Supplementary Materials
Supplementary Table

Gut microbiota	Anti-proliferative activity (%)		Gut microbiota	Anti-proliferative activity (%)	
	HCT116	CT26		HCT116	CT26
GM01	57.7 ± 2.3	15.0 ± 3.4	GM12	47.4 ± 1.5	10.5 ± 4.9
GM02	51.5 ± 1.2	15.5 ± 4.8	GM13	51.8 ± 1.3	17.2 ± 6.7
GM03	33.8 ± 4.4	-4.9 ± 3.0	GM14	61.5 ± 1.7	35.9 ± 3.5
GM04	58.8 ± 1.2	20.2 ± 1.1	GM15	65.2 ± 2.4	15.1 ± 2.3
GM05	76.7 ± 3.1	7.4 ± 3.1	GM16	61.9 ± 0.7	0.7 ± 4.0
GM06	40.2 ± 2.0	4.4 ± 2.5	GM17	78.8 ± 3.8	11.7 ± 4.6
**GM07**	**40.2 ± 0.4**	**50.5 ± 1.5**	GM18	68.1 ± 0.4	-9.9 ± 16.0
GM08	66.9 ± 0.3	22.1 ± 7.0	GM19	52.3 ± 2.4	-3.9 ± 16.7
GM09	65.4 ± 1.0	5.5 ± 12.9	GM20	57.1 ± 0.7	22.9 ± 8.2
GM10	60.2 ± 1.6	35.2 ± 5.7	GM21	56.5 ± 1.7	24.9 ± 8.2
GM11	33.4 ± 2.4	16.2 ± 9.1			

Table S1. Screening of anti-proliferative activity by cell-free supernatant (CFS) of gut microbiota. The anti-proliferative activity was measured in the precense of 21 species's CFS by MTT assay.

Supplementary Figure

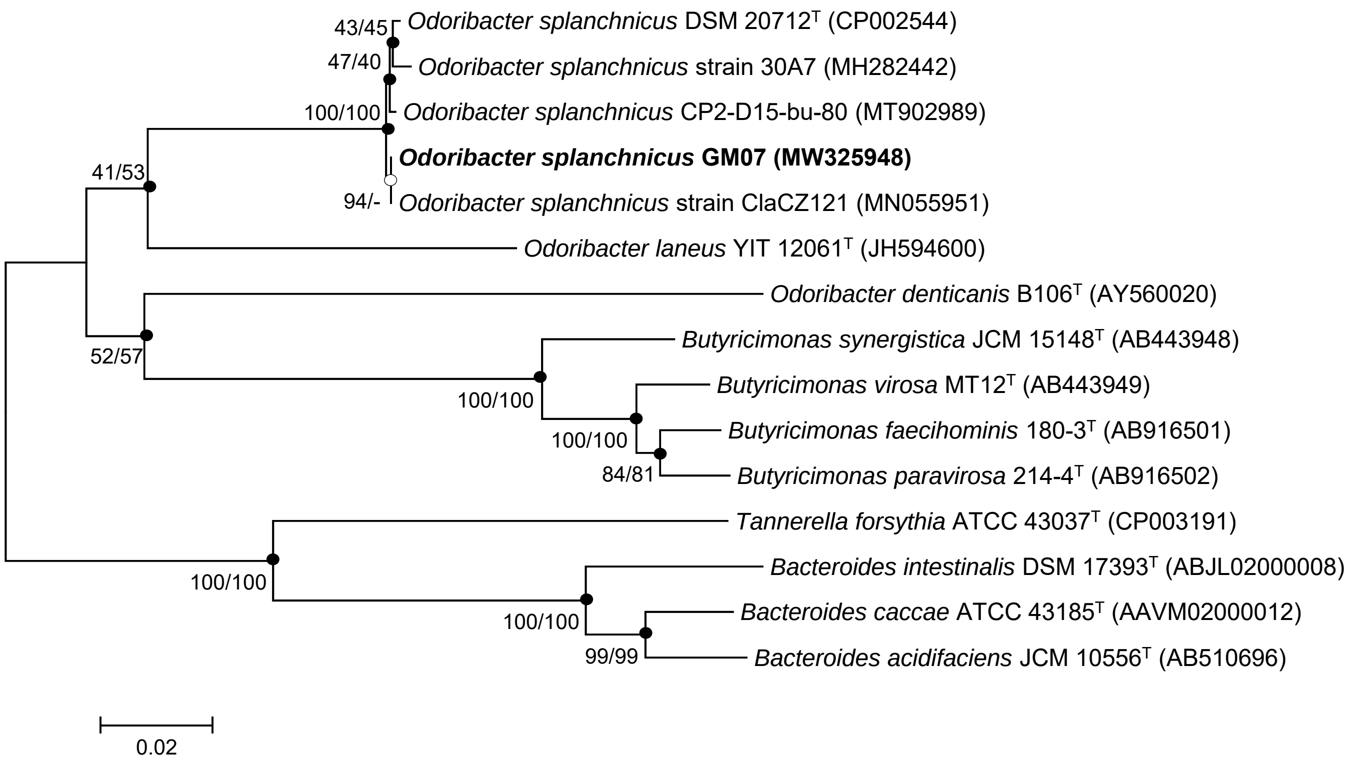

Figure S1. Phylogenetic tree showing the position of *Odoribacter splanchnicus* GM07, other *O. splanchnicus* strains and closely related species belonging to the order *Bacteroidales*, based on 16S rRNA gene sequence similarity data. The tree was merged with neighbor-joining (NJ) and maximum-likelihood (ML) algorithm. Numbers at nodes refer to bootstrap values (based on 1000 replicates, only values >50% are shown at branch points). Filled circles indicate that the corresponding nodes (groupings) were recovered by the NJ and ML methods. The bar represents 2% of sequence divergence.

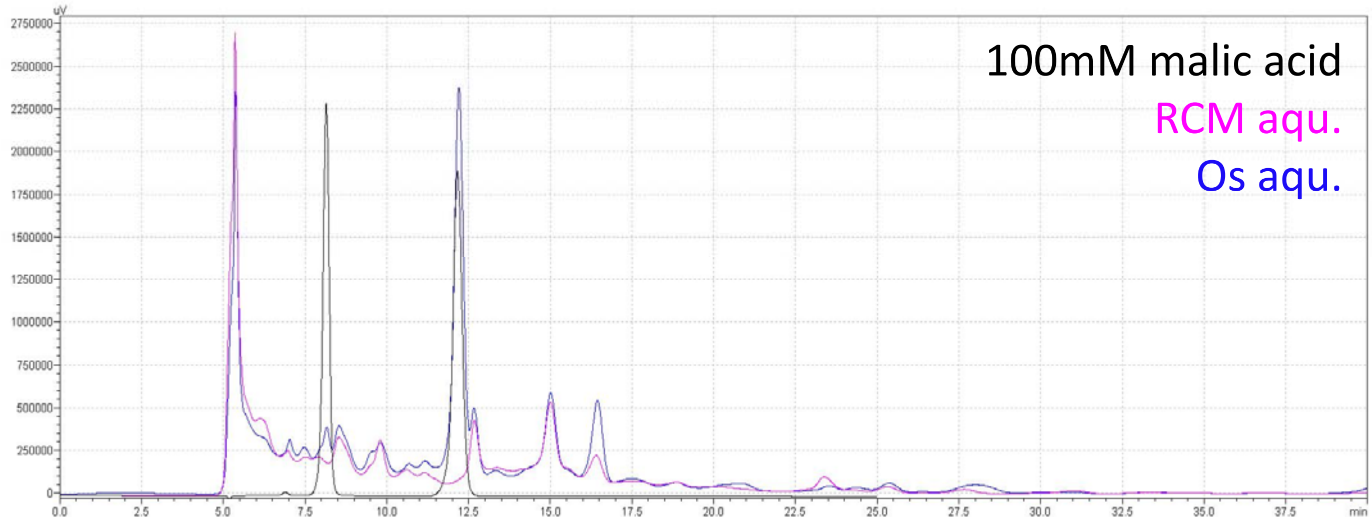

Figure S2. HPLC chromatograms of 100 mM standard of malic acid (black), the aqueous phase of RCM extract (pink) and the aqueous phase of OsCFS extract (blue).

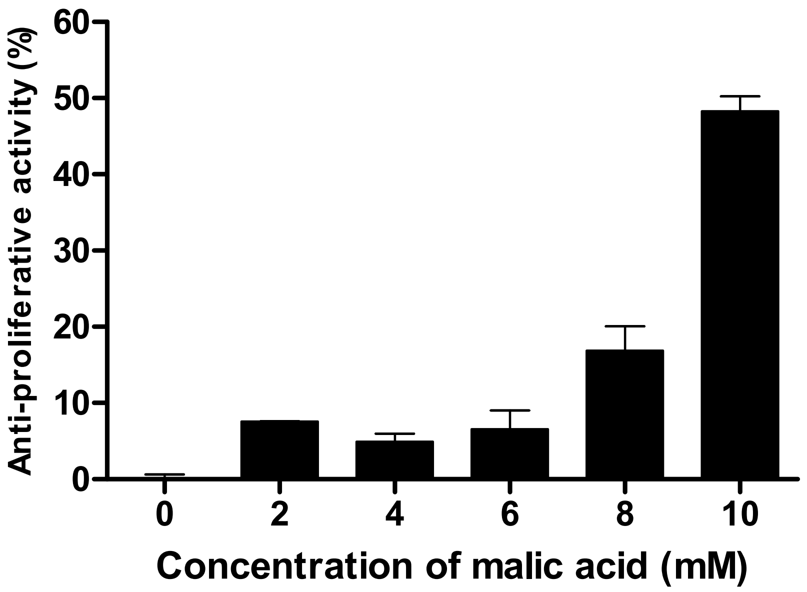

Figure S2. The anti-proliferative activity of malic acid on HCT 116 cells.
